# Tolerability and Blinding of Transcranial Direct Current Stimulation in People with Parkinson’s Disease: A Critical Review

**DOI:** 10.3390/brainsci10070467

**Published:** 2020-07-20

**Authors:** Craig D. Workman, Alexandra C. Fietsam, Thorsten Rudroff

**Affiliations:** 1 Department of Health and Human Physiology, University of Iowa, Iowa City, IA 52242, USA; alexandra-fietsam@uiowa.edu (A.C.F.); thorsten-rudroff@uiowa.edu (T.R.); 2 Department of Neurology, University of Iowa Hospitals and Clinics, Iowa City, IA 52242, USA

**Keywords:** tDCS, tolerability, blinding, Parkinson’s disease

## Abstract

Transcranial direct current stimulation (tDCS) is accompanied by transient sensations (e.g., tingling, itching, burning), which may affect treatment outcomes or break the blinding of the study protocol. Assessing tolerability and blinding is integral to providing ample evidence of a “real effect” from the applied stimulation and dispelling the possibility of placebo effects. People with Parkinson’s disease (PwPD) endure many motor and non-motor symptoms that might be amenable to tDCS. However, because the disease also affects sensation capabilities, these subjects might report tolerability and blinding differently than other cohorts. Therefore, the purpose of this review was to aggregate the tolerability and blinding reports of tDCS studies in PwPD and recommend a standard tolerability and blinding reporting practice. A literature search of the PubMed and Scopus databases from 1 January 2020 to 1 April 2020 was performed to identify publications that applied tDCS to PwPD. Seventy studies were potentially reviewable, but only 36 (nine with quantitative tolerability reports, 20 with qualitative tolerability reports, and seven that only reported blinding) provided sufficient information to be included in the review. Quantitative information on tDCS tolerability and blinding maintenance in PwPD is scarce, and future reviews and metanalyses should carefully consider the possibility of placebo effects in their included studies.

## 1. Introduction

Transcranial direct current stimulation (tDCS) involves applying electrical currents through the scalp to alter cortical excitability [1] and facilitate neural plasticity. This neuromodulation tool is an appealing therapeutic adjunct because it has a relatively low cost, is easy to administer, and has a potential for in-home use [2]. The tDCS subjects often report feeling transient sensations (e.g., tingling, itching, burning) [3], which may affect treatment outcomes by distracting them from the study task or breaking the blinding of the study protocol. Indeed, assessing both the tolerability and blinding efficacy of a given protocol is vital to the interpretation of the researched outcome and integral to providing ample evidence of a “real effect”, as opposed to a placebo effect, from the applied stimulation. To help maintain blinding, most studies apply sham tDCS, typically consisting of the administration of a short bout (≤ 1 min) of current at the beginning of the stimulation period, which purportedly provides the same sensations as active tDCS without altering cortical excitability [4]. This type of sham application has previously been shown to effectively blind subjects at intensities ≤1 mA [5,6]. However, because the most common tDCS intensity has increased from 1 mA to 2 mA [7], blinding maintenance [8,9] and controlling for placebo effects have become increasingly difficult challenges for researchers.

People with a variety of health statuses have received tDCS under diverse experimental conditions [7,10]. Although there may be some utility in applying tDCS as an ergogenic aid in healthy subjects [11,12], this is increasingly investigated as an accessory therapy for treatment-resistant symptoms in neurological and neuropsychiatric populations [13,14]. For example, people with Parkinson’s disease (PwPD) endure a host of motor (e.g., resting tremor, rigidity, bradykinesia, postural instability, gait disturbance, freezing of gait) [15,16] and non-motor (e.g., fatigue, pain, depression, sleep disturbance, bowel or bladder dysfunction) [17] symptoms, some of which are medication-resistant, which impair their independence and quality of life [18]. Several studies have investigated tDCS as an adjunct treatment for some of these refractory symptoms with some efficacy [19,20]. However, given that tolerability and blinding efficiency are likely influenced by the unique characteristics of individual subjects (e.g., age) [21] and different pain sensation capabilities (e.g., hyperalgesia) [22,23] among diverse subject populations, it is important to clarify these concepts in independent clinical populations. Therefore, the purpose of this review was to aggregate the tolerability and blinding reports of tDCS studies in PwPD from the previous decade, with the goal of updating the tolerability and blinding efficacy information of tDCS-PD research at large and recommending a standard tolerability and blinding reporting practice for future investigations.

## 2. Methods

### 2.1. Literature Search

A record search of the PubMed and Scopus databases was performed to identify publications that applied tDCS to PwPD. The search terms for titles, keywords, and abstracts were “tdcs” OR "transcranial direct current stimulation" AND "Parkinson’s disease", which were the same for both databases. The inclusion criteria were: (1) English-language studies, (2) human subjects, (3) published between 1 January 2010 and 1 April 2020, (4) and applied direct current brain stimulation to the scalp. Exclusion criteria were: (1) other forms of transcranial electrical stimulation (e.g., transcranial alternating current stimulation, transcranial random noise stimulation, etc.), deep brain stimulation, and repetitive transcranial magnetic stimulation studies (rTMS, theta burst). Initially, the “human subjects” limitation was applied to the database results (i.e., on the website) to remove non-human subject studies; however, it was observed that this limitation wrongly removed some human subject studies from the PubMed search results and that this criteria was accomplished at the title or abstract screening phase instead. Because the goal of this review was to gather as much tDCS tolerability and blinding information in PwPD as possible, brief reports, letters to the editor, conference proceedings, and abstracts were also included, provided that full text was available or retrievable via interlibrary loan to the University of Iowa libraries.

### 2.2. Screening

Database search results were examined by one reviewer (CDW) to identify studies that fit the inclusion criteria. The potentially pertinent records were exported from the database searches and examined by the same reviewer to determine if the publication was appropriate for further assessment. The bibliographies of retrieved records, whether included in the final review or not, were also searched for additional publications. The text of these potential records was examined for tolerability or adverse events and blinding information. Studies that reported either quantitative tolerability or blinding (sensation tolerability score, *p*-value, or percent/number of subjects) and qualitative (e.g., “no adverse events were reported by any of the subjects”) information were retained for final review. Studies were organized by those that reported quantitative tolerability information (either alone or with blinding information), qualitative tolerability information, and those that reported blinding information only (quantitative and qualitative). Study characteristics (e.g., design, stimulation parameters, stimulation time) were considered as potential explanations for unusual tolerability or blinding results in the reviewed studies.

## 3. Results

**Figure 1:**
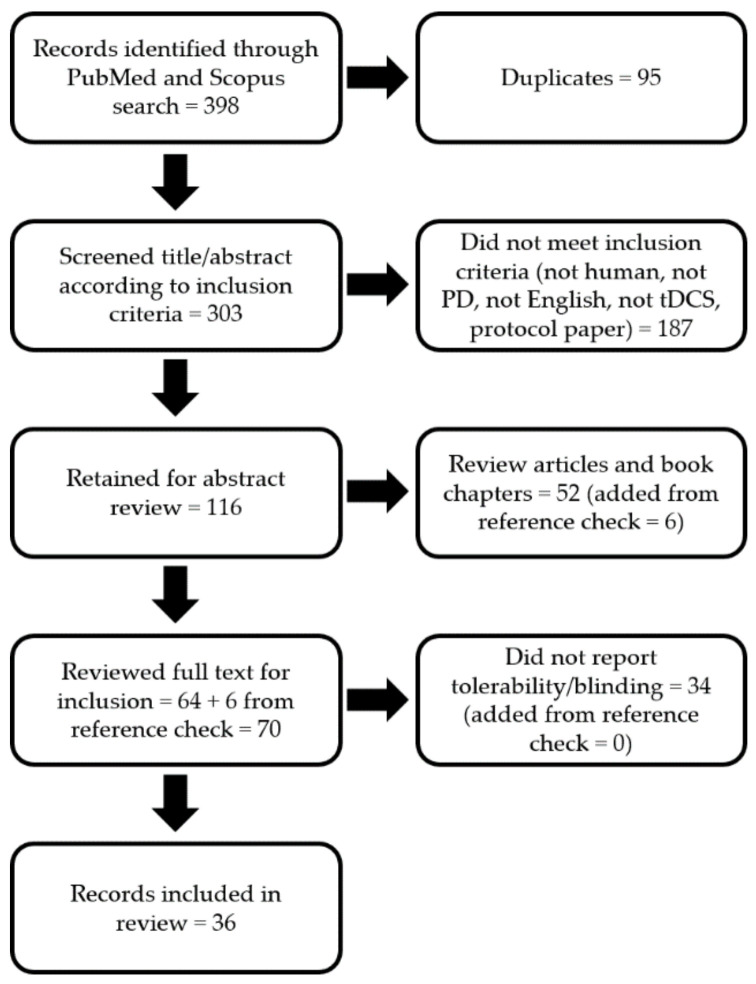
Flow chart of the literature search, screening, and study inclusion. PD = Parkinson’s disease, tDCS = transcranial direct current stimulation.

The database searches yielded 398 total citations. Of these, 95 were duplicates, 187 did not meet the inclusion or exclusion criteria (i.e., not PD, not human, not English, not tDCS, or was a protocol paper), and 52 were removed as reviews (six additional records were found by checking the references of these reviews). Accordingly, the full text of 70 publications were reviewed for full inclusion. Of these, 34 did not report any tolerability or blinding information at all and were, therefore, not reviewable. Thus, 36 studies were included for the final review (Figure 1).

Only 9 out of 36 studies provided quantitative tolerability or blinding information [24,25,26,27,28,29,30,31,32] (Table 1). Most reported the percentage or number of subjects that experienced a stated sensation [24,26,27,28,29,30,31,32], but only five indicated sensation severity [24,25,29,31,32] and only three discussed blinding [24,25,26]. Study subjects had mild to moderate PD (Hoehn and Yahr (H&Y) range = 1.6–2.5). Two studies tested patients off dopaminergic medication [26,30] and three did not report medication status [24,27,32]. The study designs were heterogeneous: two studies were open-label (no subject or researcher blinding) [28,29]; three were parallel arm, randomized, double-blind, sham-controlled designs [25,27,32]; and four were crossover, randomized, sham-controlled designs, with two being double-blind [24,26], one being single-blind (subject) [31], and one with unstated blinding [30]. All but one study administered tDCS with intensities ≤ 2 mA (range of current densities = 0.03 mA/cm^2^ – 0.08 mA/cm^2^) for 15–25 min (mode = 20 min) and targeted frontal brain areas (i.e., the dorsolateral prefrontal cortex (DLPFC), frontal polar area) [25,26,27,28,29,30,31,32]; the other study used 2 and 4 mA intensities for 20 min and targeted the cerebellum [24]. The sensations reported the most often were tingling (8/9 studies), burning (5/9 studies), and itching (4/9 studies). The severities (scaled from 1 (low) to 10 (high)) of these common sensations were mild (range = 1.0–2.8), although one study only reported that all sensations were ≤ 6 [32]. Rarer sensations (e.g., headache, pain or pressure, poking) tended to have higher severity ratings (range = 2.9–6.0) [24,29], which may coincide with the relatively high current densities applied in these studies (0.08 mA/cm^2^ and 0.11 mA/cm^2^). However, similar rare or severe sensations were reported in sham treatment and at a 0.06 mA/cm^2^ current density [24], which might be attributed to the unique stimulation location of that study (i.e., cerebellum) rather than the stimulation parameters. All three of the studies that provided a description of the maintenance of blinding integrity [24,25,26] indicated that blinding integrity was maintained, but one did not provide any quantitative blinding information [26].

Most of the reviewed studies (20/36) provided qualitative tolerability reports [33,34,35,36,37,38,39,40,41,42,43,44,45,46,47,48,49,50,51,52] (Table 2). Most included simple statements (e.g., “no adverse events were reported by any of the [subjects]” [42]), but a few provided descriptions of the experiences of individual subjects who had more severe or unique (at least for the study) adverse effects [33,40,50]; only one study mentioned blinding integrity [33]. Study subjects had mild to moderate PD (H&Y range = 1.3–3.0). One study tested in both off and on dopaminergic medication [33], one tested in the off state [49], and one did not report medication status [52]. Five investigations had open-label designs [38,43,45,46,47]; seven were parallel arm, randomized, double-blind, sham-controlled designs [33,40,41,42,44,50,52]; and eight were crossover, randomized, sham-controlled designs, with six being double-blind [36,37,39,48,49,51] and two with unstated blinding [34,35]. The tDCS intensities ranged from 1 to 2.8 mA (range of current densities = 0.02 mA/cm^2^–0.12 mA/cm^2^) and stimulation was applied for 7–30 min (mode = 20 min). Anodal targets included motor areas (e.g., unihemispheric or bihemispheric M1) and frontal areas (e.g., DLPFC). Of the three studies with more detailed reports [33,40,50], one had an unusual two-cathode configuration (over both mastoids) and attributed the reported adverse event (i.e., “small first degree burns”) to poorly positioned cathodal electrodes in one subject [33]. Another was stimulated for 30 min with a 2-mA intensity, which may have contributed to the reported burning sensation under the centralized supraorbital cathodes of two subjects [50]. The third study did not have any unusual stimulation parameters that might explain the reported tingling and light flash experienced by one subject and the reported event might be attributed to the individual characteristics of that subject or slightly different electrode placement in that session [40]. Lastly, although the group sizes and characteristics were not identical, the possibility of at least some of the subjects being included in all three of the Hadoush et al. papers is noted [45,46,47], and the results of Grüner et al. [34] and Eggers et al. [35] were explicitly stated to be from the same subjects.

The remaining seven studies either only reported blinding information or discussed tolerability with the purpose of verifying blinding integrity, and all studies indicated successful subject blinding [53,54,55,56,57,58,59] (Table 3). All study subjects had mild to moderate PD (H&Y range = 1.8–2.5). All but one study testing patients on dopaminergic medication, while the remaining study did not report medication status [59]. One was a parallel arm, randomized, double-blind, sham-controlled design [54]; and the rest were crossover, randomized, sham-controlled designs, with three double-blinded [55,58,59] and three single-blinded studies (subject) [53,56,57]. The intensities of these studies ranged from 1 to 2.8 mA (range of current densities = 0.03–0.08 mA/cm^2^) and was applied for 6–20 min (mode = 20 min). Anodal targets included unihemispheric and bihemispheric frontal areas (DLPFC, frontal polar area) and M1. Lastly, it was noted that the two articles by Broeder et al. had identical subject characteristics, z-scores, and *p*-values, and might represent blinding data from the same subjects [56,57].

## 4. Discussion

The purpose of this review was to combine and critically review the tolerability and blinding reports of tDCS studies in PwPD from the previous decade and to update the tolerability and blinding efficacy status of tDCS-PD research. The notable findings were that (1) nearly half (34/70) of the potentially reviewable studies were excluded for not reporting any tolerability or blinding information (Figure 1); (2) the majority of reviewed studies mentioned tolerability (29/36; Table 1 and Table 2), but only nine provided quantitative information (percentage or number of subjects), five of which also reported sensation severity scores (Table 1); (3) only 11 out of 36 studies mentioned blinding integrity, with eight providing quantitative (e.g., *p*-value, percentage) information (three in Table 1, one in Table 2, and all in Table 3). Altogether, this review highlights a stark underreporting of quantitative tolerability (9/70 = 12.9%; 5/70 = 7.1% via severity score; 4/70 = 5.7% via percentages) and blinding (8/70 = 11.4%) in tDCS-PD research in the past decade.

As discussed above, investigating the tolerability and blinding of tDCS is essential to defining the efficacy of this technique as an adjunct intervention. Because so few studies reported sufficient tolerability or blinding information, deciding the efficacy of tDCS in PwPD, although outside of the scope of this review, would be impractical. Furthermore, despite the considerable evidence that tDCS affects cortical excitability, as measured by transcranial magnetic stimulation (TMS) [60], without more comprehensive tolerability and blinding information, one cannot exclude placebo effects as a contributor to any positive outcomes from tDCS. For example, Petersen and Puthusserypady [61] recently showed brain activity alterations when subjects donned a tDCS device and were told they were receiving stimulation during a cognitive task. Even though the device never delivered any stimulation in the sham condition (not even a stimulation ramp-up), the authors found significant alterations in electroencephalography (EEG) signals in the placebo group compared with the control group. Similarly, a definitive link between motor evoked potential (MEP) increase or decrease and performance alteration is still uncertain (see Abdelmoula et al. [62] and Lopez-Alonso et al. [63] for examples).

Despite using the same stimulation parameters on all subjects in each study, the tolerance of stimulation-related sensations is highly subjective and individual subjects might report different sensations and acceptance of those sensations in diverse ways. PD is more common in older adults and might have a higher prevalence in men [64], which is reflected in the age and sex distributions of the subjects in many of the reviewed studies. Thus, some of this individuality of responses might also be affected by age [21], sex [65], or comorbidities, which warrant future investigations. Furthermore, the rare but sometimes more severe severities reported in a few of the reviewed studies [24,29] also support this individuality notion. Nevertheless, sensation tolerability in different subject populations is a meaningful topic to explore because clinical populations (e.g., PD, multiple sclerosis, stroke) might be more or less prone to reporting severe sensations based on the etiology and symptomatology of their disease (e.g., hypoalgesia vs. hyperalgesia). Tolerability also helps define the safety of tDCS, and some safety reviews have used sensation reports as part of their safety definition [66,67]. In addition, many previous safety and tolerability reviews have operated under the assumption that an absence of tolerability reporting is evidence that no subjects reported any sensations or that the sensations were unremarkable (see Bikson et al. [7] and Antal et al. [68] for examples). Although this inference might be valid, a better and more complete understanding of sensation tolerability and tDCS safety would be realized via systematic reporting of the presence or absence of sensations and their severity by tDCS researchers.

The placebo effect found by Petersen and Puthusserypady [61] discussed above is in opposition to a review and meta-analysis that indicated no effect of sham tDCS on cortical spinal excitability [6]. Still, these findings raise an important question regarding the placebo effects of standard sham paradigms, especially if blinding is not preserved. Currently, the most prevalent tDCS intensities have increased from 1 to 2 mA [7] and might go beyond 2 mA [69] in populations that might have a theoretically increased benefit from higher intensity stimulation, such as PD (see the preliminary results of Workman et al. [24] for an example). Given that blinding maintenance is less feasible with intensities ≥ 2 mA [8,9], particularly in non-naïve subjects [70], placebo effects pose a potential threat to the validity of performance outcomes in tDCS studies. Therefore, alternative sham methods, such as a 30 s ramp-up followed by 30 s to 1 min of stimulation before a 30 s ramp-down to 0 mA [71,72] or sensation attenuation via topical analgesics [73] might be required to maintain blinding integrity, which warrant systematic investigation.

There are several limitations to note for this review. First, the article search was restricted to English language articles only, which potentially decreased the number of studies that could have been included in the review. Second, studies that did not report any tolerability or blinding information were excluded from full review. Although it might be reasonable to assume (as others have done [7,68]) that these excluded studies did not have any notable tolerability effects to describe (e.g., “no adverse effects were reported”), their inclusion would have been counterproductive to the purpose of this review.

## 5. Summary and Recommendation

Only a minority of potentially reviewable studies (17/70 = 24.3%) reported quantitative tolerability or blinding information, and most of the reviewed records only provided qualitative tolerability or blinding statements (21/36 = 58.3%). Thus, quantitative information on tDCS tolerability and blinding maintenance is scarce. In the absence of this information, future reviews and meta-analyses should carefully consider the possibility of placebo effects in their included studies. Furthermore, at a minimum it is recommended that future tDCS studies should collect tolerability information for each sensation (e.g., visual analogue scale (VAS) or 10 point scale) and blinding information (sham or active guesses, confidence in guess (VAS or 10 point scale)) for each subject. Other potential reporting options could involve enquiring where the sensations are occurring (at the anode, cathode, both sites, or whole head) [74] and the time course of stimulation-related sensations [24] (Appendix A provides a recommended tolerability and blinding data collection form). The results of these scales could also be subjected to appropriate statistical testing to determine differences between sessions or study groups. Without the minimum information to inform tolerability and blinding integrity, researchers cannot have full confidence that their tDCS outcomes were not influenced by placebo effects.

## Figures and Tables

**Table 1:** Reviewed studies that reported quantitative sensation information (sensation scores, *p*-values, percentages of subjects). Studies that also provided blinding information (*n* = 3) are listed first. Data are means ± SD.

Citation	Sample (n, Age, Men/Women, H&Y Med Status)	Design	^a^ Montage (Electrode Size)	Intensity (Density)	Duration (Timing, Task)	^b^ Summary
Workman et al. [24]	7, 72.4 ± 6.4, 5/2, 1.9 ± 0.4 NR	Crossover, randomized, double-blind, sham (single session)	a: cerebellum c: cerebellum (bilateral) OR upper arm of most PD-affected side (unilateral) (35 cm^2^)	2 mA (0.06 mA/cm^2^) 4 mA (0.11 mA/cm^2^)	20 min (Offline)	^d^**Sham**: Tingling (14.3%, 1.5 ± 0.71), itching (28.6%, 2.17 ± 0.41) burning (42.86%, 1.33 ± 0.58), pins/needles (28.5%, 2.5 ± 0.4), poking (14.3%, 4.0 ± 0.0). Guessed sham (0%), 2 mA (71.4%), 4 mA (28.6%). **Unilateral 2 mA**: Tingling (57.1% 1.4 ± 0.0), itching (14.3%, 2.0 ± 0.0), burning (14.3%, 1.0 ± 0.0), pins/needles (28.6%, 2.5 ± 1.2), tickling (28.6%, 1.5 ± 1.0), prickling (14.3%, 1.0 ± 0.0). Guessed sham (42.9%), 2 mA (57.1%), 4 mA (0%). **Bilateral 2 mA**: Tingling (28.6%, 1.8 ± 1.2), itching (14.3%, 2.0 ± 0.0), burning (28.6%, 2.3 ± 1.5), pins/needles (14.3%, 3.0 ± 0.0), poking (14.3%, 6.0 ± 0.0), prickling (14.3%, 3.0 ± 0.0). Guessed sham (28.6%), 2 mA (57.1%), 4 mA (14.3%). **Unilateral 4 mA**: Tingling (28.6%, 1.3 ± 0.4), burning (28.6%, 1.5 ± 0.7), pins/needles (42.9%, 2.1 ± 1.2), poking (14.3%, 5.0 ± 0.0), tickling (14.3%, 2.0 ± 0.0), prickling (14.3%, 1.0 ± 0.0). Guessed sham (14.3%), 2 mA (71.4%), 4 mA (14.3%). **Bilateral 4mA:** Tingling (14.3%, 2.0 ± 0.0), itching (14.3%, 1.3 ± 0.0), burning (57.1%, 2.4 ± 0.2), pins/needles (28.6%, 3.5 ± 0.8), tickling (14.3%, 1.0 ± 0.0). Guessed sham (0%), 2 mA (57.1%), 4 mA (42.9%).
Manenti et al. [25]	Active: 11, 65.5 ± 6.4, 5/6, 1.6 ± 0.8 On Sham: 11, 63.8 ± 7.1, 7/4, 1.9 ± 0.5 On	Parallel, randomized, double-blind, sham (10 sessions over 2 weeks)	a: F3 c: cSO (35 cm^2^)	2 mA (0.06 mA/cm^2^)	25 min (Online; CT)	Since the scores reported in the active group (session 1 (1.18 ± 0.72); session 10 (1.00 ± 0.60)) were comparable with the scores in the sham group (session 1 (1.09 ± 0.79); session 10 (1.27 ± 1.05, *p* = 0.49)), there were no reasons to reject the double-blinded character of this study. No adverse effects were reported.
Lau et al. [26]	10, 62.7 ± 6.6, 5/5, 2.2 ± 0.3 Off	Crossover, randomized, double-blind, sham (single session)	a: F3 c: cSO (35 cm^2^)	2 mA (0.06 mA/cm^2^)	20 min (Online, WM, Inhibition tasks)	In total, 80% experienced initial tingling sensation from both active and sham. All tolerated the intervention without pain or major discomfort. None were able to distinguish between active and sham.
Doruk et al. [27]	18, 61 ± 8, 12/6, NR NR	Parallel, randomized, double-blind, sham (10 sessions over 2 weeks)	a: F3 or F4 c: cSO (35 cm^2^)	2 mA (0.06 mA/cm^2^)	20 min (Offline)	Tingling (50%), sleepiness (55%), mild headache (22%), neck pain (11%), skin redness (22%), and trouble concentrating (22%). None reported unexpected or severe adverse effects.
Shaw et al. [28]	6, NR, NR, NR On	Open label (10 sessions over 2 weeks)	a: l-DLPFC c: r-DLPFC (NR)	2 mA (unknown)	20 min (Online; CT)	^c^ Tingling (41.3%), itching (7.7%), burning (30.8%), headache (4.8%)
Dobbs et al. [29]	16, 66.9 ± 5.4, 13/3, NR On	Open label (10 sessions over 2 weeks)	a: F3 c: F4 (25 cm^2^)	2 mA (0.08 mA/cm^2^)	20 min (Online; CT)	Tingling (43%, 2.2), itching (8%, 2.6), burning (29%, 2.4), headache (6%, 2.7), localized head pain/pressure (8%, 2.9), difficulty concentrating (1%, 1)
Ishikuro et al. [30]	9, 77.5 ± 4.8, 3/6, 1.9 ± 0.6 Off	Crossover, randomized, sham (5 sessions over 1 week)	a: FPA or OPA c: OPA or FPA (35 cm^2^)	1 mA (0.03 mA/cm^2^)	15 min (Offline)	Five (55.6%) felt mild tingling. No other adverse effects observed.
Putzolu et al. [31]	FoG+: 10, 70.1 ± 3.84, 6/4, NR On FoG-: 10, 72.8 ± 6.87, 5/5, NR On	Crossover, randomized, single-blind, sham (single session)	a: F3 c: cSO (25 cm^2^)	1.5 mA (0.06 mA/cm^2^)	20 min (Offline)	Active: Tingling or burning (75%, 2.8 ± 0.25). Sham: Tingling or burning (68%, 2.6 ± 0.36).
Sharma et al. [32]	Group n = 17 Active: NR, 65.3 ± 7.7, NR, 2.5 ± 0.4 NR Sham: NR, 66.2 ± 6.1, NR, 2.3 ± 0.5 NR	Parallel, randomized, double-blind, sham (10 sessions over 2 weeks) Offered an additional 10 open-label sessions	a: F3 c: F4 (NR)	2 mA (unknown)	20 min (Online, CT)	Tingling (22.4%), itching (8.2%), burning (11.5%), headache (3.3%), nausea (0.9%), dizziness (0.3%), sleepiness (0.3%). All pain ratings ≤ 6.

^a^ Electrode locations are either from the 10-20 or 10-10 electroencephalography standard, or the stated brain region. ^b^ Severity reports scaled from 1 (low) to 10 (high). ^c^ Includes subjects with multiple sclerosis. ^d^ Sensation data calculated as mean of means and mean of SDs across reported time points (beginning, middle, end). PD = Parkinson’s disease, Med status: on or off dopaminergic medication when tested; H&Y: Hoehn and Yahr scale; a: anode; c: cathode; NR: not reported; cSO: contralateral supraorbit; l: left; r: right; CT: cognitive training; FPA: frontal polar area; OPA: occipital area; FoG: freezing of gait; WM: working memory.

**Table 2:** Reviewed studies that reported qualitative sensation information.

Citation	Sample (n, Age, Men/Women, H&Y Med Status)	Design	^a^ Montage (Electrode Size)	Intensity (Density)	Duration (Timing, task)	Summary
Benninger et al. [33]	Active: 13, 63.6 ± 9.0, 9/4, 2.5 ± 0.1 Off/On Sham: 12, 64.2 ± 8.8, 7/5, 2.4 ± 0.2 Off/On	Parallel, randomized, double-blind, sham (8 sessions over 2.5 weeks)	a: 10 mm anterior to Cz or center forehead (97.5 cm^2^) c: mastoids (25 cm^2^; two cathodes)	2 mA (a: 0.02 mA/cm^2^)	20 min (Offline)	Small first degree burns likely caused by accidentally poorly positioned electrodes over the mastoids partially covering the earlobes in a single subject (completely healed within 3 days). No other adverse events. All subjects experienced occasional, short-duration “tingling”, but no pain or discomfort. Blinding appeared reliable based on patients’ and blinded raters’ reports.
Grüner et al. [34]	15, 68.6 ± 8.2, 9/6, 2.5 ± 0.5 On	Crossover, randomized, sham (single session)	a: M1 or cSO c: cSO or M1 (35 cm^2^)	1 mA (0.03 mA/cm^2^)	10 min (Offline)	All tolerated well without side-effects.
Eggers et al. [35]	15, 68.6 ± 8.2, 9/6, 2.5 ± 0.5 On	Crossover, randomized, sham (single session)	a: M1 or cSO c: cSO or M1 (35 cm^2^)	1 mA (0.03 mA/cm^2^)	10 min (Offline)	All subjects tolerated the stimulation session well and without side-effects. Note: same subjects, conditions, and sessions as Grüner et al. [34]
Manenti et al. [36]	10, 67.1 ± 7.2, 6/4, 1.3 ± 1.1 On	Crossover, randomized, double-blind, sham (single session)	a: F3 or F4 c: cSO (35 cm^2^)	2 mA (0.06 mA/cm^2^)	7 min (Offline)	Inferred that all subjects tolerated the stimulation well.
Valentino et al. [37]	10, 72.3 ± 3.6, 5/5, 2.8 ± 0.5 On	Crossover, randomized, double-blind, sham (5 sessions over 1 week)	a: M1 c: cSO (NR)	2 m (unknown)	20 min (Offline)	The experimental procedures were well-tolerated and no adverse effects were observed. All subjects reported a tingling or itching sensation over the electrode placement area only at the beginning and at the end of the stimulation, without differences between sham and anodal tDCS.
Elder et al. [38]	8, 64.63 ± 8.16, 7/1, NR On	Open label (single session)	a: 50% between F3 and FP1 c: right deltoid muscle (35 cm^2^)	2.8 mA (0.08 mA/cm^2^)	20 min (Offline)	^b^ All participants tolerated stimulation and did not report any side effects, other than a brief tingling sensation under the electrodes, during or immediately after stimulation. No adverse events were reported.
Cosentino et al. [39]	16, 66.9 ± 5.4, 8/8, NR On	Crossover, randomized, double-blind, sham (single session)	a: M1 or cSO c: cSO or M1 (25 cm^2^)	2 mA (0.08 mA/cm^2^)	20 min (Offline)	The experimental procedures were well-tolerated and no adverse effects were reported by any of the subjects.
Schabrun et al. [40]	Active: 8, 72.0 ± 4.9, 8/0, 2 ± 0 On Sham: 8, 63.0 ± 11.0, 2/6, 2 ± 0 On	Parallel, randomized, double-blind, sham (9 sessions over 3 weeks)	a: l-M1 c: cSO (35 cm^2^)	2 mA (0.06 mA/cm^2^)	20 min (Online; GT)	One participant experienced strong tingling over the site of one electrode and a momentary flash of light. The sensations lasted approximately 5 s. No other events or symptoms reported.
Chang et al. [41]	Active: 16, 63.6 ± 7.5, 9/7, 2.5 ± 0.6 On Sham: 16, 63.8 ± 8.3, 11/5, 2.4 ± 0.5 On	Parallel, randomized, double-blind, sham (5 sessions over 1 week)	a: F3 c: cSO (25 cm^2^)	1 mA (0.04 mA/cm^2^)	20 min (Offline)	All subjects completed the study with no significant adverse effects.
Costa-Ribeiro et al. [42]	Active: 11, 61.1 ± 9.1, 8/3, 2.4 ± 0.7 On Sham: 11, 62.0 ± 16.7, 7/4, 2.3 ± 0.7 On	Parallel, randomized, double-blind, sham (10 sessions over 4 weeks)	a: 2 cm anterior to Cz c: SO contralateral to more-affected side (35 cm^2^)	2 mA (0.06 mA/cm^2^)	13 min (Offline)	No adverse events were reported by any of the subjects.
Agarwal et al. [43]	16, 67.6 ± 5.9, 13/3, 2.0 ± 0.1 On	Open label (10 sessions over 2 weeks)	a: l-DLPFC c: r-DLPFC (25 cm^2^)	2 mA (0.08 mA/cm^2^)	20 min (Online; CT)	All sessions (100%) were tolerated and completed successfully.
da Silva et al. [44]	Active: 8, 66 ± 5, 4/4, 2.3 ± 0.4 On Sham: 9, 66 ± 10, 6/3, 2.4 ± 0.2 On	Parallel, randomized, double-blind, sham (single session)	a: 1.8 cm anterior to Cz c: SO ipsilateral to more-affected side (35 cm^2^)	2 mA (0.06 mA/cm^2^)	15 min (Offline)	No subjects reported adverse events associated with the stimulation session.
Hadoush et al. [45]	18, 62.1 ± 9.5, 13/5, 2.7 ± 0.9 On	Open label (10 sessions over 2 weeks)	a: FC1 & FC2 c: cSO & cSO (25 cm^2^)	1 mA (0.04 mA/cm^2^)	20 min (Offline)	This stimulation dose and protocol had no adverse effects.
Hadoush et al. [46]	21, 62.5 ± 9.0 15/6, 3.0 ± 0.8 On	Open label (10 sessions over 2 weeks)	a: FC1 & FC2 c: cSO & cSO (25 cm^2^)	1 mA (0.04 mA/cm^2^)	20 min (Offline)	All subjects completed the study with no reported side effects.
Bueno et al. [48]	20, 64.5 ± 9.0, 12/8, 2.3 ± 0.6 On	Crossover, randomized, double-blind, sham (single session)	a: F3 c: cSO (35 cm^2^)	2 mA (0.06 mA/cm^2^)	20 min (Offline)	All subjects demonstrated good tolerability toward the application of the stimulation without exhibiting any adverse effects.
Lu et al. [49]	10, 62.1 ± 9.5 7/3, 2.7 ± 0.9 Off	Crossover, randomized, double-blind, sham (single session)	a: SMA, ~1.8 cm anterior to Cz (8.1 cm^2^ butterfly electrode) c: Center forehead (51 cm^2^)	1 mA (a: 0.12 mA/cm^2^)	10 min (Offline)	All subjects completed the study and no adverse events were reported.
Yotnuengnit et al. [50]	tDCS: 18, 64.4 ± 7.8 10/8, 2.4 ± 0.5 On PT: 18, 62.7 ± 8.8 12/6, 2.4 ± 0.5 On Combo: 17, 68.2 ± 9.8 11/6, 2.5 ± 0.5 On	Parallel, randomized, double -blind, sham (6 sessions over 2 weeks)	a: Cz c: central SO (35 cm^2^)	2 mA (0.06 mA/cm^2^)	30 min (Offline & Online; PT)	During the intervention period, two subjects, who received the anodal tDCS intervention for the first time, reported a burning sensation on their forehead where the electrode was attached. As the day progressed, this subsided without any treatment. For the subsequent anodal tDCS interventions, more water was added to the electrodes and the two patients did not experience recurrence of the symptom.
Putzolu et al. [51]	FoG+: 10, 69.20 ± 5.20, 6/4, 2.05 ± 0.44 On FoG-: 11, 70.36 ± 6.23, 7/4, 1.77 ± 0.52 On	Crossover, randomized, double-blind, sham (single session)	a: F3 c: FP2 (25 cm^2^)	1.5 mA (0.06 mA/cm^2^)	20 min (Offline)	No adverse tDCS-related events were noted. No adverse tDCS-related events were observed at any the testing times.
Elder et al. [52]	^b^ Active: 19, 76.3 ± 8.8, 15/4, NR NR Sham: 17, 73.9 ± 7.0, 12/5, NR NR	Parallel, randomized, double-blind, sham (2 sessions per day, 4 days total)	a: P4 c: Oz (25 cm^2^)	1.2 mA (0.05 mA/cm^2^)	20 min (Offline)	All subjects tolerated stimulation. Other than a brief tingling sensation underneath the electrodes, no adverse events were reported.

^a^ Electrode locations are either from the 10-20 or 10-10 electroencephalography standard, or the stated brain region. ^b^ Includes subjects with Lewy body dementia. PD = Parkinson’s disease, Med status: on or off dopaminergic medication when tested; H&Y: Hoehn and Yahr scale; a: anode; c: cathode; l: left; r: right; NR: not reported; cSO: contralateral supraorbit; M1: primary motor cortex; GT: gait training; DLPFC: dorsolateral prefrontal cortex; CT: cognitive training; SMA: supplementary motor area; WM = working memory; PT: physical therapy; FoG: freezing of gait.

**Table 3:** Reviewed studies that reported blinding integrity verification.

Citation	Sample (n, Age, Men/Women, H&Y Med Status)	Design	^a^ Montage (Electrode size)	Intensity (Density)	Duration (Timing, task)	Summary
von Papen et al. [53]	10, 64 ± 10, 3/7, NR On	Crossover, randomized, single-blind, sham (single session)	a: M1 c: cSO (35 cm^2^)	1 mA (0.03 mA/cm^2^)	10 min (Offline)	None of the participants was able to discriminate sham from real tDCS.
Manenti et al. [54]	Active: 10, 69.0 ± 9.1, 4/6, 2.2 ± 0.6 On Sham: 10, 69.1 ± 5.6, 7/3, 2.3 ± 0.4 On	Parallel, randomized, double-blind, sham (10 sessions over 2 weeks)	a: DLPFC c: cSO (35 cm^2^)	2 mA (0.06 mA/cm^2^)	25 min (Online; PT)	The scores reported in the active group were comparable with the scores in the sham group (*t* = −0.90, *p* = 0.40), such that the two could not be distinguished. Hence, there were no reasons to reject the double-blinded character of this study.
Elder et al. [55]	38, 66.6 ± 8.4, 27/11, NR On	Crossover, randomized, double-blind, sham (single session)	a: l-DLPFC c: r-deltoid (35 cm^2^)	2.8 mA (0.08 mA/cm^2^)	20 min (Offline)	No adverse events were reported, and participants were blinded to stimulation condition (*p* > 0.05).
Broeder et al. [56]	10, 63.2 ± 9.2, 8/2, ^b^2.0 (2.0, 2.0) On	Crossover, randomized, single-blind, sham (single session)	a: F3 c: cSO (35 cm^2^)	1 mA (0.03 mA/cm^2^)	20 min (Online; WT)	No adverse events of tDCS were reported. There was no significant difference between the VAS scores after tDCS and sham stimulation (z = 1.332, *p* = 0.183).
Broeder et al. [57]	10, 63.2 ± 9.2, 8/2, ^b^2.0 (2.0, 2.0) On	Crossover, randomized, single-blind, sham (single session)	a: M1 c: cSO (35 cm^2^)	1 mA (0.03 mA/cm^2^)	20 min (Online; WT)	There were no dropouts, and no adverse events occurred. Comparing VAS scores after tDCS and sham revealed no significant difference between conditions in either group (*z* = 1.332; *p* = 0.183).
Dagan et al. [58]	20, 68.8 ± 6.8, 17/3, 2.5 ± 0.6 On	Crossover, randomized, double-blind, sham (single session)	a: Cz or Cz/F3 c: AF4, CP1, FC1 or AF4, CP1, FC1, FC5 (pi-electrodes 3 cm^2^)	1.5 mA (0.6 mA/cm^2^)	20 min (Offline)	After multitarget and after M1-only stimulation, ≥70% of the subjects believed that they received real stimulation, with similar confidence levels ^b^ (6.43 and 7.19, respectively). Following sham, 50% of the participants thought they received real stimulation, with a relatively high confidence level (7.6). When comparing the 3 stimulations, no significant difference was found in the number of subjects who reported real or sham. The confidence levels were also similar after real and sham responses.
Adenzato et al. [59]	20, 65.6 ± 8.4, 10/10, 1.8 ± 0.7 NR	Crossover, randomized, double-blind, sham (single session)	a: FPz c: Between inion and Oz (35 cm^2^)	1.5 mA (0.04 mA/cm^2^)	6 min (Online; CT)	Responses to the sensation’s questionnaire completed by patients with PD-MCI at the end of each stimulation session revealed that all of the subjects tolerated the stimulation well. A Wilcoxon matched pairs test revealed that perceptual sensations reported after the active and sham stimulation sessions were not significantly different (T = 13.5, z = 1.73; *p* = 0.08). Thus, there was no reason to reject the blinded nature of this study.

^a^ Electrode locations are either from the 10-20 or 10-10 electroencephalography standard, or the stated brain region. ^b^ Median (1st quartile, 3rd quartile). PD = Parkinson’s disease, Med status: on or off dopaminergic medication when tested; H&Y: Hoehn and Yahr scale; a: anode, c: cathode; cSO: contralateral supraorbit; DLPFC: dorsolateral prefrontal cortex; PT: physical therapy; l: left; r: right; CT: cognitive task; WT = writing task; VAS: visual analogue scale for pain.

## Appendix A

**Table A1:** Ask which stimulation condition they think they received and how confident they are in their guess. Ask about sensations experienced at different time points, their severity, and location. Administer immediately after stimulation.

Condition: __________________			Confidence:						
1	2	3	4	5	6	7	8	9	10
Not confident at all					Completely confident				
Felt at the **BEGINNING** of the Stimulation									
Sensation and severity: __________________ Location: anode/cathode/both/whole head									
1	2	3	4	5	6	7	8	9	10
Barely perceptible					Most I could possibly stand				
Sensation and severity: __________________ Location: anode/cathode/both/whole head									
1	2	3	4	5	6	7	8	9	10
Barely perceptible					Most I could possibly stand				
Felt in the **MIDDLE** of the Stimulation									
Sensation and severity: __________________ Location: anode/cathode/both/whole head									
1	2	3	4	5	6	7	8	9	10
Barely perceptible					Most I could possibly stand				
Sensation and severity: __________________ Location: anode/cathode/both/whole head									
1	2	3	4	5	6	7	8	9	10
Barely perceptible					Most I could possibly stand				
Felt at the **END** of the Stimulation									
Sensation and severity: __________________ Location: anode/cathode/both/whole head									
1	2	3	4	5	6	7	8	9	10
Barely perceptible					Most I could possibly stand				
Sensation and severity: __________________ Location: anode/cathode/both/whole head									
1	2	3	4	5	6	7	8	9	10
Barely perceptible					Most I could possibly stand				
